# Advances in the Application of Biomimetic Endometrium Interfaces for Uterine Bioengineering in Female Infertility

**DOI:** 10.3389/fbioe.2020.00153

**Published:** 2020-02-28

**Authors:** Qixin Han, Yanzhi Du

**Affiliations:** ^1^Center for Reproductive Medicine, Ren Ji Hospital, School of Medicine, Shanghai Jiao Tong University, Shanghai, China; ^2^Shanghai Key Laboratory for Assisted Reproduction and Reproductive Genetics, Shanghai, China

**Keywords:** endometrium interface, uterus regeneration, nano-scale, biomimetic scaffold, female infertility

## Abstract

The Asherman’s syndrome, also known as intrauterine adhesion, often follows endometrium injuries resulting from dilation and curettage, hysteroscopic resection, and myomectomy as well as infection. It often leads to scarring formation and female infertility. Pathological changes mainly include gland atrophy, lack of vascular stromal tissues and hypoxia and anemia microenvironment in the adhesion areas. Surgical intervention, hormone therapy and intrauterine device implantation are the present clinical treatments for Asherman’s syndrome. However, they do not result in functional endometrium recovery or pregnancy rate improvement. Instead, an increasing number of researches have paid attention to the reconstruction of biomimetic endometrium interfaces with advanced tissue engineering technology in recent decades. From micro-scale cell sheet engineering and cell-seeded biological scaffolds to nano-scale extracellular vesicles and bioactive molecule delivery, biomimetic endometrium interfaces not only recreate physiological multi-layered structures but also restore an appropriate nutritional microenvironment by increasing vascularization and reducing immune responses. This review comprehensively discusses the advances in the application of novel biocompatible functionalized endometrium interface scaffolds for uterine tissue regeneration in female infertility.

## Introduction

Secondary infertility is the most common type of female infertility worldwide, often because of endometrium injuries and subsequent intrauterine adhesion (IUA). It poses a great threat to female physical and mental health (Deans et al., 2018; Vander Borght and Wyns, 2018; Dreisler and Kjer, 2019). The uterus tissue is made up of three layers, among which the endometrium, composed of functional and basal layers, is the inner-most layer. The functional endometrium is the site of embryo implantation and is regulated by changes in ovarian hormones. The basal endometrium regenerates and repairs the endometrium wound after menstruation, and forms the functional layer again, possibly via the intrinsic endometrial cells, such as endometrial epithelial and stromal cells, endometrial stem cells and perivascular cells. They may secrete bioactive molecules, growth factors, hormones and contribute to angiogenesis and endometrium regeneration after uterus injuries. The pathological changes include endometrial fibrosis and scarring, loss or thinning of endometrium due to different degrees of damage to the basal layer of endometrium, IUA between anterior and posterior walls, and shrinkage of uterine cavity (Conforti et al., 2013). Microscopic observation shows gland atrophy, lack of vascular stromal tissues and hypoxia and anemia microenvironment in the adhesion areas (Evans-Hoeker and Young, 2014; Healy et al., 2016). Present clinical techniques, such as hormonal therapy, surgical synechiotomy and subsequent intrauterine device (IUD) implantation, show unsatisfactory outcomes, recurrent adhesion and secondary infection during the treatment of IUA, also known as Asherman’s syndrome (Cai et al., 2016, 2017; Mo et al., 2019). The surgical synechiotomy helps surgeons release the adhesive fibrosis with blunt-end scissors. However, the postoperative recovery shows a huge variation among different patients due to adhesion severity. Some of them even experienced greater adhesion recurrence. Hormonal therapy works effectively after surgical release of IUA. Nevertheless, it is still hard to confirm a suitable medication dosage and route due to the short half-life period, low water solubility and big differences in response. As for the IUDs, they only function as physical barriers. However, they can barely induce regenerative process and thus yield low endometrium recovery. Therefore, it is urgent and vital to find alternative treatments for Asherman’s syndrome (Dreisler and Kjer, 2019).

**FIGURE 1:**
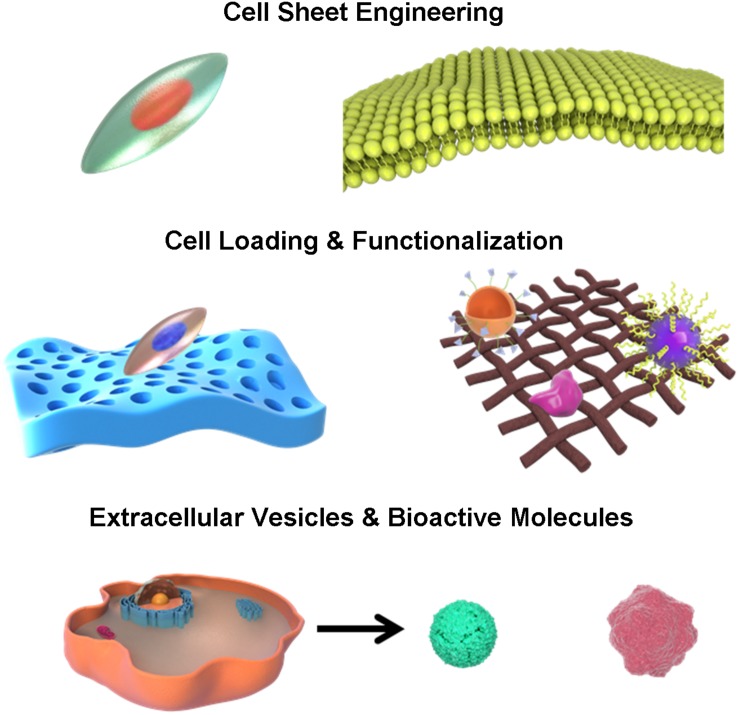
Schematic illustration of different biomimetic endometrium interfaces manufacturing.

The development of biomimetic tissue engineering provides an alternative therapy that may increase the success of uterine regeneration and reproductive capacity (Cervelló et al., 2015). Biomaterial is an important factor in the tissue engineering because it can provide structural support that mimics native endometrium tissues and uterine organs (Zhang et al., 2020). In addition, some biomedical materials are characterized by physical, chemical and biological properties that are closely related to uterus regeneration. The other two factors in the tissue engineering are supporting cells and bioactive molecules. They both facilitate cellular and extracellular signaling, nutrient transport, stem cell recruitment, proliferation and differentiation. Biomaterials can release drugs, growth factors, small molecules and other bioactive compounds in a controlled style, with or without cell loading and modification. Recent researches have shown that, in addition to traditional biomaterial based uterus regeneration, combination and modification of cells and biomaterials, such as cell sheets, cell-scaffold interfaces, surface-functionalized scaffolds and decellularization of biological tissues may also display functional or structural advantages and repair injured uterus to different extents by inducing biomimetic changes and recreating regenerative microenvironment (Liu et al., 2019a). Therefore, we comprehensively reviewed current advances in the biological interactions and applications of different types of biomimetic endometrium and uterus scaffolds for female infertility treatment and compared their potential therapeutic effects in this review (Figure 1).

## Stem Cell Sheet Engineering in the Endometrium Regeneration

Cell sheet engineering (CSE) is an emerging technique for cell transplantation using temperature-responsive culture dishes to repair damaged organs or tissues and has displayed potential capacity in the tissue regeneration (Okano et al., 1993). Cell sheets naturally have two sides: apical and basal interfaces. Both of them contained abundant extracellular matrix (ECM) necessary for providing maximum strength and adherence to the host tissue surface (Wang X. et al., 2018). CSE includes cell culture in thermo-sensitive plates that develop compact cell sheets which detach with temperature drop (Jun et al., 2017). As a major advantage, this technology permits maintenance of growth factors, ECM proteins, and other bioactive cytokines because cells are detached spontaneously from the culture plates without the need for enzymatic procedures and proteolytic treatments. The endometrial epithelial cell is a local cell type in the endometrium of uterus. It is suitable to seek these cells for cell engineering (Kuramoto et al., 2018). However, in real practices, the collection of endometrial cells is not a recommended option because it is hard to collect adequate quantity of endometrial cells from the uterus and collection may also be interfered by certain inflammatory or contagious diseases, apart from the harm out of invasive procedures (Logan et al., 2018). Stem cell sheet engineering (SCSE) gradually becomes an important research direction due to the minimal invasiveness, autologous tissue supply, multi-differentiation potential, high proliferation, growth factor secretion and signaling transduction of stem cells (Shum et al., 2017; Hsu et al., 2019; Imafuku et al., 2019).

Oral mucosal epithelial cell sheets (OMECS) were applied in IUA prevention and subsequent infertility as a novel regenerative technique (Kuramoto et al., 2015). OMECS were obtained by minimal invasiveness surgery from oral mucosal epithelial tissues and were composed of stratified squamous epithelial cells. Therefore, cell collection was easy and harmless. OMECS successfully retained the luminal structure of the uterine and prevented re-adhesion occurrence by controlling neutrophil infiltration.

Although the thermo-responsive approach is very effective, it is further modified and improved due to the high cost, and time-consuming properties. Sun et al. (2018) developed a new and user-friendly method by adding ascorbic acid into adipose-derived stem cells (ADSC) to construct cell sheets. ADSCs are widely used for tissue engineering due to their self-renewable, proliferative and regenerative characteristics (Mao et al., 2019; Rogan et al., 2020). After *in vitro* culture, ADSCs secreted and were surrounded by ECM proteins. The expansion of cell sheet constructs further increased expression of many ECM proteins and trophic growth factors including vascular endothelial growth factor (VEGF) and fibroblast growth factor (FGF) (Yu et al., 2018). The implantation of ADSC sheets in the damaged rat uterus prevented luminal stenosis or scar formation by decreasing transforming growth factor-β (TGF-β) and collagen I levels, similar to sham operation. The cell sheets stimulated blood vessel development and endometrial gland proliferation in the endometrial stromal layer. In contrast, the blank control group displayed hyperplastic fibrosis. ADSCs were successfully differentiated into endometrial stromal cells under uterine microenvironment. The findings indicated implantable ADSC sheets attached closely to the injured uterine and facilitated endometrium repair by providing a biomimetic trophic support that was vital for cell proliferation. Natural cell sheet constructs did not induce any inflammation and thus caused insignificant scar formation (Sun et al., 2018). SCSE is very beneficial for uterus repair by secreting growth factors and nutritional proteins. However, the cell viability remains a serious question *in vivo* and requires better scaffold support for long-term survival (Table 1).

**TABLE 1:** Fabrication and functions of different biomimetic scaffolds for endometrium and uterus regeneration.

**Interface type**	**Cells**	**Construct technique**	**Biological effects**	**Model**	**Author/year**
Cell sheet	OMEC	Thermo-sensitive plate	Maintenance of luminal structure, little neutrophil infiltration	F344/NJcl-rnu/rnu rats	Kuramoto et al., 2015
Cell sheet	ADSC	Fusion of ascorbic acid	Trophic factor release, angiogenesis, no inflammation	SD rats	Sun et al., 2018
Cell-scaffold	dEMSC	*In vitro* decidualization and encapsulation	Shortening recovery time, better mimicking native tissues and stiffness	C57BL/6 mice	Kim Y. Y. et al., 2019
Cell-scaffold	En-PSC	CYR61-transfected cell loading	Increasing the blood vessel density and angiogenic growth factor	SD rats	Li Z. et al., 2019
Cell-scaffold	BMSC	Cell seeding on collagen	Stem cell recruitment and microvasculature regeneration	SD rats	Ding et al., 2014
Cell-scaffold	BMSC	Encapsulation by thermo-responsive gelation of PF-127 and vitamin C	Redox homeostasis, endometrial thickness recovery	SD rat	Yang et al., 2017
Cell-scaffold	BMSC	Solvent casting and particle leaching	Improving BMSC proliferation and differentiation, *in situ* retention, vascularization	SD rats	Xiao et al., 2019
Cell-scaffold	BMNC	Cell seeding on collagen	Downregulating ΔN p63 expression and inhibiting endometrial quiescence	Human patients	Zhao et al., 2017
Cell-scaffold	UCMSC	Cell seeding on collagen	Endometrial proliferation, differentiation and vascularization	Human patients	Cao et al., 2018
Cell-scaffold	UCMSC	Cell mixture with collagen fibers	Collagen deposition and reduced scar formation	SD rats	Xu L. et al., 2017
Cell-scaffold	UCMSC	Freeze-drying and thermal treatment crosslinking	Increasing estrogen, progesterone and growth factor levels; epithelial reconstruction	SD rats	Xin et al., 2019
Cell-scaffold	hESC	Cell seeding on collagen	High differentiation ratio	SD rats	Song et al., 2015
Functionalized scaffold	/	Fermentation and lipholization	Vascularization and endometrium maturation	SD rats	Cai H. et al., 2019
Functionalized scaffold	/	CBD-modified bFGF	Target delivery of bFGF and prolonging retention time	SD rats	Li et al., 2011
Functionalized scaffold	/	CBD-modified VEGF	Activation of MMP and remodeling of ECM	SD rats	Lin et al., 2012
Functionalized scaffold	/	Droplet microfluidics	Steady bFGF release, no side effects or excessive loss of burst delivery	SD rats	Cai Y. et al., 2019
Functionalized scaffold	/	Sol-gel transition	Increasing autophagy by inhibition of mTOR signaling pathway	SD rats	Xu H. L. et al., 2017
Functionalized scaffold	/	Lipholization and rotary evaporation	Increasing vascularity by activation of ERK1/2 pathways	SD rats	Zhang et al., 2017
Functionalized scaffold	/	Ultraviolet irradiation and gel formation	Increasing regeneration-related cytokines and prolonging secretome retention	SD rats	Liu et al., 2019b
Decellularized scaffold	/	Aortic perfusion with detergents	Preservation of native ECM and vasculature	SD rats	Miyazaki and Maruyama, 2014
Decellularized scaffold	/	Decellularization by detergents or high hydrostatic pressure	Collagen retention, uterine tissue repair combined with cell migration	SD rats	Santoso et al., 2014
Decellularized scaffold	/	Perfusion with Triton, dimethyl sulfoxide or sodium deoxycholate	Recellularization of the scaffold and infiltration of regional stem cells	Lewis rats	Hellström et al., 2016
Decellularized scaffold	/	Whole organ perfusion	Successful recellularization by human side population stem cells	/	Campo et al., 2017
Decellularized scaffold	/	Perfusion with 0.25% and 0.5% SDS and preservation in 10% formalin	Biomimetic mechanical, structural, and angiogenic characteristics	Wistar rats	Daryabari et al., 2019
Decellularized scaffold	/	Perfusion with SDS	Orientation of smooth muscle layers and ECM	SD rats	Miki et al., 2019

*OMEC, oral mucosal epithelial cell; ADSC, adipose-derived stem cell; dEMSC, decidualized endometrial stromal cell; En-PSC, endometrial perivascular cell; CYR61, cysteine-rich angiogenic inducer 61; BMSC, bone marrow mesenchymal stem cell; BMNC, bone marrow mononuclear cell; UCMSC, umbilical cord derived mesenchymal stem cell; hESC, human embryonic stem cell; CBD, collagen binding domain; bFGF, basic fibroblast growth factor; MMP, matrix metalloprotein; ECM, extracellular matrix; mTOR, mammalian target of rapamycin; ERK 1/2, extracellular regulated protein kinases 1/2; SDS, sodium dodecyl sulfate; SD, Sprague Dawley. “/” means “not applicable.”*

## Cell-Scaffold Interface-Based Endometrium Regeneration

Biomaterials provide structural and mechanical support for tissue repair by helping to restore the architecture and functionality of the wounded tissues. They may also partially mimic native environment by inducing physicochemical changes and simulating changes of growth factors, signaling molecules and extracellular vesicles like cellular materials (Kim S. et al., 2019; Liu et al., 2019a). However, it is insufficient to simply use scaffold biomaterial for repairing large uterus defects (Xin et al., 2019). Several factors should be considered carefully, such as vascularization, native cell recruitment, and scar inhibition (Owusu-Akyaw et al., 2019). Cell seeding on the scaffold materials increases biological functions by prolonging cell survival and stimulating cell proliferation, differentiation and vascularization (Frost et al., 2019).

Kim Y. Y. et al. (2019) also used decidualized endometrial stromal cells (dEMSCs) encapsulated in hyaluronic acid (HA) hydrogel in a murine uterine infertility model. At 2 weeks after injury, the fibrous tissues were decreased and the endometrium thickness was increased. Some embryonic markers, including desmin, CD44, and platelet endothelial cell adhesion molecule, were highly expressed and secreted in the functional regenerated endometrium. Successful implantation of transferred embryos was followed by normal development and live birth of offspring after the dEMSC-loaded HA hydrogel treatment. The selection of bioprocessed isotopic cells shortened the recovery time significantly compared to bone marrow mesenchymal stem cells (BMSCs) or human embryonic stem cell (hESC)-derived endometrium-like cells. In addition, HA appeared to be very suitable for the repair of endometrium where plenty of hyaluronidase could degrade HA. Furthermore, the limiting mobility of cross-linkage with porosity allowed seeding cells to maintain in the injured site and provided the ideal scaffold stiffness for endometrium regeneration.

Li Z. et al. (2019) focused on the restoration of angiogenesis and inhibition of scar tissue formation in the selection of cell types for uterine repair. Endometrial perivascular cells (CD146 + platelet derived growth factor receptor (PDGFR) β +) (En-PSCs) worked similarly as stem cells in the endometrial layer. Cysteine-rich angiogenic inducer 61 (CYR61) contributed to vascular formation (Zhao et al., 2018). They thus designed a CYR61-transfected En-PSC-loaded collagen scaffold and found it significantly increased the blood vessel density because the scaffold stimulated the release of angiogenic factors from the ECM and accelerated an overall process of neovascularization *in vivo*.

Apart from endometrium-derived cells, BMSCs were largely used for endometrium and uterus regeneration due to their convenient isolation, abundant resources and reparative potential (Xia et al., 2019). Ding et al. (2014) transplanted BMSC-loaded collagen scaffolds to the wounded rat uterine horn. BMSCs were mainly recruited at the regenerated basal membrane of the endometrium. The injured tissue next to the cell-scaffold composite showed high expression levels of basic FGF (bFGF), insulin-like growth factor 1 (IGF-1), TGF-β1, VEGF and prominent microvasculature regeneration. BMSC-loaded scaffold recovered the receptive ability of the new endometrium. Yang et al. (2017) reported that BMSCs were encapsulated by thermo-responsive gelation of pluronic F-127 (PF-127) and vitamin C, which added to the membrane stability. In addition, vitamin C, as a prominent antioxidant, downregulated tumor necrosis factor α (TNF-α) and interleukin 6 (IL-6) secretion, maintained redox homeostasis and facilitated a pro-regenerative tendency by increasing interleukin 10 (IL-10) level (El Banna et al., 2019; Qi et al., 2019). The BMSC/PF-127 + vitamin C hydrogel recovered endometrial thickness and decreased the fibrotic regions of the stromal tissues of endometrium. Xiao et al. (2019) loaded BMSCs on a synthetic Poly(glycerol sebacate) (PGS) scaffold which potentially recovered different deformations of soft tissues in various dynamic conditions without external irritations. They compared different cell-scaffold constitutes, including BMSC/collagen, BMSC/poly(lactic-co-glycolic acid) (PLGA), and BMSC/PGS. They found PGS showed a stronger improvement in proliferation and endometrial differentiation of BMSC. Furthermore, *in vivo* studies showed a longer *in situ* retention time of BMSC and higher areas of vascularization from PGS-based scaffold. It was vital for successful recuperation of damaged uterus tissues. Bone marrow mononuclear cells (BMNCs) are derived from hematopoietic stem cells in the bone marrow and develop in the bone marrow. Zhao et al. (2017) applied collagen scaffolds loaded with BMNCs in patients of Asherman’s syndrome by downregulating ΔNp63 expression and inhibiting endometrial quiescence and other related pathological changes. BMNC-loaded collagen scaffolds restored estradiol (E2) stimulation and reaction to functional endometrium growth. Five patients were successfully pregnant and delivered live births.

Umbilical cord derived mesenchymal stem cells (UCMSCs) have displayed their merits in adequate sources, pain-free acquisition and excellent proliferation capacity (Lee et al., 2019). UCMSCs were loaded on collagen scaffolds and transferred to injured uterus of human patients. Cao et al. (2018) confirmed an improvement in endometrial proliferation, differentiation and neovascularization following the implantation of this cell-scaffold mixture without introducing exogenous DNA to the regenerated endometrium. Furthermore, most babies were born without any obvious birth defects or placenta complications. Xu L. et al. (2017) also focused on UCMSCs and their potentials for reducing scar formation. They found UCMSCs mixed with gelatinous degradable collagen fibers showed prominent angiogenesis and insignificant scarring in the injured site. The cell-scaffold composite degraded collagen in scarring areas by increasing matrix metalloprotein 9 (MMP-9), FGF-2 and VEGF and led to angiogenesis and endometrial cyclic regeneration. Xin et al. (2019) found UCMSC-loaded collagen scaffold reduced cellular apoptosis and improved human endometrial stromal cell via a paracrine route. The scaffold barely caused any inflammation because it contributed to collagen remodeling in the reconstructed endometrium. In addition, UCMSC-loaded collagen scaffold induced early rapid re-epithelialization by increasing proliferation and cytokeratin expression levels, which were vital for subsequent endometrium repair after damages. The scaffold subsequently elevated circulating estrogen and progesterone levels as well as growth factor expression.

Song et al. (2015) loaded hESC-derived endometrium-like cells on collagen scaffolds to repair uterine horn damages. They innovatively induced endometrial differentiation by adding endometrial stromal cells and achieved a differentiation rate of above 80%, 2.2 fold higher than the cytokine induction. Large quantities of endometrium-like cells improved endometrial function and development by simulating an *in vivo* endometrium stem cell niche, and secreted growth factors that modulated the effects of estrogen and progesterone-driven basal layer repair. Cell loading on the scaffolds improves the biological activity of biomaterials and keeps the physiochemical properties to support the mechanical stability of tissue regrowth (Table 1).

## Surface Functionalized Scaffold-Based Endometrium Regeneration

In addition to direct cell loading in the scaffolds, many strategies are focused on the surface or structure modification for better biocompatibility and stronger absorption for cell attachment and delivery of bioactive growth factors, hormones and extracellular vesicles (Li C. et al., 2019; Shadish et al., 2019). Bacterial cellulose (BC) is a biocompatible and water adsorbable bacteria scaffold. It was used in bone, vessel and nerve repair (Huang et al., 2017; Wang B. et al., 2018; Rebelo et al., 2019). Cai H. et al. (2019) improved the BC porosity by supplementing silk fibroin (SF) and stromal cell derived factor 1 α (SDF-1α). The functionalized nanoscaffold delivered SDF-1α from SF-BC membrane carrier and induced uterine cell migration *in vitro* and increased endometrium thickness and number of fetuses. They explained the effects of functionalized BC scaffold as it specifically improved migration and regeneration of glandular epithelial cells, which were vital for decidualization, implantation, and embryo development. In addition, the scaffold significantly increased arterial formation. These findings were mainly due to the dual effects of SDF-1α loaded SF-BC scaffolds: vascularization and endometrium maturation.

Li et al. (2011) designed a collagen scaffold loaded with collagen binding domain (CBD)-modified bFGF. This combination significantly reduced the random diffusion of bFGF *in vivo* and increased target delivery at the endometrium. The recombinant proteins were transported in a location specific style with collagen and kept the effective concentration in the injured area. The complex scaffold induced high neovascularization, muscle fiber alignment and thick endometrium layers, which was very effective to tissue repair. However, the embryo rate was low in this study, indicating the functional recovery of the endometrium was not achieved.

Similarly, Lin et al. (2012) loaded CBD/VEGF on the collagen scaffold for improving angiogenesis and endometrium re-epithelialization. They compared different release manners of VEGF, including CBD and native injection, in the regeneration of full-thickness injury of rat uterus. The vascular tissue growth provided the scar areas with nutrients and oxygen. Furthermore, target release of VEGF activated MMP and initiated ECM remodeling by increasing inflammatory cells at early stages. The findings showed a 31.2% improvement of pregnancy rate in the application of CBD/VEGF collagen (50.0%) compared with local VEGF injection only (18.8%).

Cai Y. et al. (2019) invented a new method for bFGF controlled release because of the porous surface and external–internal based open porous architecture. They fabricated the bFGF-loaded porous scaffold from microfluidic droplets. The adjustable porous design could be controlled precisely. It facilitated steady bFGF release and avoided side effects and excessive loss of burst delivery in high concentrations. The long-term reparative performance was excellent due to adhesion inhibition, vascular promotion and induction for endothelial cell migration by this bFGF-loaded porous scaffold.

Xu H. L. et al. (2017) fabricated a temperature sensitive hydrogel loaded by keratinocyte growth factor (KGF), a kind of reparative factor. The scaffold allowed controlled release and prolonged retention of the drug in the injured uterus. They found KGF-modified hydrogel scaffold facilitated cell autophagy by inhibition of mammalian target of rapamycin (mTOR) signaling pathway and improved CD31 expression levels, endothelial migration and proliferation of endometrial glandular epithelial cells and luminal epithelial cells. Functional epithelial repair was due to restoration of appropriate micro-milieu by reducing inflammation and immune responses (Gargett et al., 2008; Zhang Z. et al., 2016).

Similarly, Zhang et al. (2017) fabricated 17β-E2-loaded heparin-poloxamer hydrogel and found it significantly decreased endoplasmic reticulum (ER) stress-related apoptosis. E2 sustained release effectively reduced fibrotic tissue areas and stimulated vascularity to provide more nutrients, oxygen, and hormones to the injured tissues, supported by activated extracellular regulated protein kinases 1/2 (ERK1/2) pathways that closely participated in some cellular activities, such as proliferation, viability, and motility (Peng et al., 2010; Matsumura et al., 2017).

In addition to bioactive proteins, some researches focused on the secreted extracellular vesicles from stem cells for uterus repair (Zhang Y. et al., 2016; Azizi et al., 2018). Liu et al. (2019b) innovatively created stem cell secretome modified-HA hydrogel that increased release of a number of regeneration-related growth factors, such as epidermal growth factor (EGF), FGF, IGF-1, and IGF binding protein (IGFBP). The crosslinked HA gel served as a carrier and prolonged the *in vivo* retention time of stem cell secretome, thus leading to thicker endometrium and more glands compared to gel application only. Nano-scale functionalization of endometrium scaffolds mimics the natural environment, provides steady release of bioactive molecules and transmits signaling from extracellular vesicles in the process of uterus regeneration (Table 1).

## Decellularized Biomimetic Scaffolds for Severe Uterine Injury Regeneration

Decellularized scaffolds are one of the alternatives for treatment of severe uterine injury because of their biocompatibility compared with synthetic material (Chen et al., 2019). These scaffold increased pregnancy and birth rate initially at the cost of long-term immunosuppressive therapy when scientists attempted in whole-organ transplantation in the early stage (Brännström et al., 2015). However, it ignited hope for decellularization of biomimetic scaffolds for severe uterine injury repair. Miyazaki and Maruyama (2014) fabricated decellularized uterine matrix scaffold from rat uterus by aortic perfusion with detergents. The scaffold provided not only mechanical support for uterine but also vascular architecture for blood perfusion. In addition, it induced recellularization, uterus regeneration and high pregnancy rate, close to the uninjured uterus. Santoso et al. (2014) used different methods for decellularization of the uterine matrix from rat uterus by sodium dodecyl sulfate (SDS) or high hydrostatic pressure (HHP), and found the latter better preserved ECM and was more efficient in cell removal. The HHP method also avoided collagen denaturation and reduction of protein contents. Interestingly, these two methods yielded entirely different manners of structural repair. In the SDS group, the repair mode was regeneration of tissue from native uterine tissue under the decellularized ECM. Nevertheless, cell migration and tissue restoration were combined as a unit in the HHP group. Hellström et al. (2016) created a uterine patch from rat uterus using perfusion method for scaffold decellularization and found the scaffold was biocompatible after recellularization *in vivo*. However, in their studies, the functional regeneration of the uterus was failed due to low pregnancy rates.

Campo et al. (2017) employed decellularization and recellularization technique in the fabrication of a scaffold from porcine uterus and displayed the excellent vascular network in the ECM after recellularization by human side population stem cells. Similarly, Daryabari et al. (2019) reported a whole-organ perfusion decellularization method for production of scaffold from ovine uterus and implanted its segments into rats. The scaffold successfully retained the vascular structure after decellularization and started recellularization in the endometrium and myometrium after implantation, potentially due to homing of the circulating and local stem cells. In addition, the excellent biomechanical properties guaranteed uterine regeneration for a long term *in vivo*. Miki et al. (2019) found orientation in the smooth muscle cells and ECM was a vital factor of correct tissue topology and functional uterine regeneration by a decellularized scaffold from rat uterus. These researches indicate that decellularized biomaterials are helpful to functional uterus regeneration, due to their biocompatibility, regulation of cell survival and homing, and topological support (Table 1).

## Discussion

Currently, there are no ideal treatments for severe IUA. Surgical release, hormone application or IUDs show their defects, respectively, such as failure in complete lysis and dosage control, and mismatch of IUD size (Salazar et al., 2017). Thus, they cannot fully repair injured endometrium and uterus. Regenerative medicine by tissue engineering offers plenty of alternative choices that may heal the wound and repair the injuries by structurally and biologically mimicking the native organ and environment (Paim et al., 2018). Recent development in various endometrium and uterus scaffolds shows promising outcomes in regard to morphological and functional recovery as well as pregnancy rates. Cell tissue engineering mainly includes cell sheets engineering and cell-scaffold interfaces. CSE is suitable for providing ECM-like elements and maintaining activity of different cytokines and growth factors. Nevertheless, it may not prolong cell proliferation and retention without appropriate scaffold materials. Therefore, surface loading of different cells on the scaffolds is widely under investigation based on *in vivo* studies that have shown rapid re-epithelialization, formation of endometrium stem cell niche and hormone-driven basal layer regeneration. In addition to cell-related engineering, structural functionalization is very important and is studied extensively. Supporting scaffold materials are composed of synthetic and natural scaffolds. In this review, we discussed surface-functionalized scaffolds and decellularized scaffolds out of biological tissues. Generally, they should exert positive influences on cellular viability, including cell proliferation, attachment and differentiation. Scaffold functionalization may contribute to endometrium repair by facilitating bioactive factor release, such as growth factors, extracellular vesicles, and other signaling molecules. Decellularization of biological tissues provides ideal collagen matrix. At the same time, it should not introduce external cellular components or cause immune rejection. However, partial cell residuals remain a non-negligible issue that prevents translational application of this technique (Destefani et al., 2017). Therefore, these different technological approaches to endometrium regeneration by tissue engineering have their merits and shortcomings that wait for further researches and investigation on possible solutions and improvement.

## Conclusion

In conclusion, there are still few efficient strategies for uterus repair in spite of current clinical solutions. Bioengineering techniques provide fresh alternatives for the traditional surgical intervention, hormone therapy and IUD implantation. The application of cell tissue constructs, cell-scaffold complex, micro- or nano- scale material release and their combination significantly enriches the therapeutic category and improves the structural and functional regeneration of injured endometrium and uterus. Future directions should be focused on the combined studies concerning cell biology and scaffold topology. Neither cells nor scaffolds alone display full recovery of endometrium and uterus and sometimes lead to low pregnancy rates. The dynamic integration of these two elements is vital for biomimetic reconstruction of physiological uterus from both structural and functional perspectives. The balanced immune milieu and angiogenic environment significantly promote tissue regeneration and organ repair. Overall, these translational approaches have enormous potential in the treatment of female infertility in the future clinical practice.

## Author Contributions

YD and QH conceptualized, designed, and wrote the manuscript. QH drafted the manuscript, reviewed the literature, and designed the figure and table. YD revised the manuscript. Both authors approved the final version of the manuscript.

## Conflict of Interest

The authors declare that the research was conducted in the absence of any commercial or financial relationships that could be construed as a potential conflict of interest.

## References

[B1] Azizi R. Aghebati-Maleki L. Nouri M. Marofi F. Negargar S. Yousefi M. (2018). Stem cell therapy in Asherman syndrome and thin endometrium: stem cell- based therapy. *Biomed. Pharmacother.* 102 333–343. 10.1016/j.biopha.2018.03.091 29571018

[B2] Brännström M. Johannesson L. Bokström H. Kvarnström N. Mölne J. Dahm-Kähler P. (2015). Livebirth after uterus transplantation. *Lancet* 385 607–616. 10.1016/S0140-6736(14)61728-1 25301505

[B3] Cai H. Li H. He Y. (2016). Interceed and estrogen reduce uterine adhesions and fibrosis and improve endometrial receptivity in a rabbit model of intrauterine adhesions. *Reprod. Sci.* 23 1208–1216. 10.1177/1933719116632923 26895816

[B4] Cai H. Qiao L. Song K. He Y. (2017). Oxidized, regenerated cellulose adhesion barrier plus intrauterine device prevents recurrence after adhesiolysis for moderate to severe intrauterine adhesions. *J. Minim. Invasive Gynecol.* 24 80–88. 10.1016/j.jmig.2016.09.021 27742483

[B5] Cai H. Wu B. Li Y. Liu Y. Shi L. Gong L. (2019). Local delivery of silk-cellulose incorporated with stromal cell-derived factor-1α functionally improves the uterus repair. *Tissue Eng. Part A* 25 1514–1526. 10.1089/ten.TEA.2018.0283 30838933

[B6] Cai Y. Wu F. Yu Y. Liu Y. Shao C. Gu H. (2019). Porous scaffolds from droplet microfluidics for prevention of intrauterine adhesion. *Acta Biomater.* 84 222–230. 10.1016/j.actbio.2018.11.016 30476581

[B7] Campo H. Baptista P. M. López-Pérez N. Faus A. Cervelló I. Simón C. (2017). De- and recellularization of the pig uterus: a bioengineering pilot study. *Biol. Reprod.* 96 34–45. 10.1095/biolreprod.116.143396 28395322

[B8] Cao Y. Sun H. Zhu H. Zhu X. Tang X. Yan G. (2018). Allogeneic cell therapy using umbilical cord MSCs on collagen scaffolds for patients with recurrent uterine adhesion: a phase I clinical trial. *Stem Cell Res. Ther.* 9:192. 10.1186/s13287-018-0904-3 29996892 PMC6042450

[B9] Cervelló I. Santamaría X. Miyazaki K. Maruyama T. Simón C. (2015). Cell therapy and tissue engineering from and toward the uterus. *Semin. Reprod. Med.* 33 366–372. 10.1055/s-0035-1559581 26285168

[B10] Chen X. Sun J. Li X. Mao L. Zhou Y. Cui L. (2019). Antifibrotic effects of decellularized and lyophilized human amniotic membrane transplant on the formation of intrauterine adhesion. *Exp. Clin. Transplant.* 17 236–242. 10.6002/ect.2017.0284 30251940

[B11] Conforti A. Alviggi C. Mollo A. De Placido G. Magos A. (2013). The management of asherman syndrome: a review of literature. *Reprod. Biol. Endocrinol.* 11:118. 10.1186/1477-7827-11-118 24373209 PMC3880005

[B12] Daryabari S. S. Kajbafzadeh A. M. Fendereski K. Ghorbani F. Dehnavi M. Rostami M. (2019). Development of an efficient perfusion-based protocol for whole-organ decellularization of the ovine uterus as a human-sized model and in vivo application of the bioscaffolds. *J. Assist. Reprod. Genet.* 36 1211–1223. 10.1007/s10815-019-01463-4 31093867 PMC6603122

[B13] Deans R. Vancaillie T. Ledger W. Liu J. Abbott J. A. (2018). Live birth rate and obstetric complications following the hysteroscopic management of intrauterine adhesions including asherman syndrome. *Hum. Reprod.* 33 1847–1853. 10.1093/humrep/dey237 30239778

[B14] Destefani A. C. Sirtoli G. M. Nogueira B. V. (2017). Advances in the knowledge about kidney decellularization and repopulation. *Front. Bioeng. Biotechnol.* 5:34. 10.3389/fbioe.2017.00034 28620603 PMC5451511

[B15] Ding L. Li X. Sun H. Su J. Lin N. Péault B. (2014). Transplantation of bone marrow mesenchymal stem cells on collagen scaffolds for the functional regeneration of injured rat uterus. *Biomaterials* 35 4888–4900. 10.1016/j.biomaterials.2014.02.046 24680661

[B16] Dreisler E. Kjer J. J. (2019). Asherman’s syndrome: current perspectives on diagnosis and management. *Int. J. Womens Health* 11 191–198. 10.2147/IJWH.S165474 30936754 PMC6430995

[B17] El Banna N. Hatem E. Heneman-Masurel A. Léger T. Baïlle D. Vernis L. (2019). Redox modifications of cysteine-containing proteins, cell cycle arrest and translation inhibition: involvement in vitamin C-induced breast cancer cell death. *Redox Biol.* 26:101290. 10.1016/j.redox.2019.101290 31412312 PMC6831881

[B18] Evans-Hoeker E. A. Young S. L. (2014). Endometrial receptivity and intrauterine adhesive disease. *Semin. Reprod. Med.* 32 392–401. 10.1055/s-0034-1376358 24959821

[B19] Frost B. A. Sutliff B. P. Thayer P. Bortner M. J. Foster E. J. (2019). Gradient poly(ethylene glycol) diacrylate and cellulose nanocrystals tissue engineering composite scaffolds via extrusion bioprinting. *Front. Bioeng. Biotechnol.* 7:280. 10.3389/fbioe.2019.00280 31681754 PMC6813186

[B20] Gargett C. E. Chan R. W. Schwab K. E. (2008). Hormone and growth factor signaling in endometrial renewal: role of stem/progenitor cells. *Mol. Cell. Endocrinol.* 288 22–29. 10.1016/j.mce.2008.02.026 18403104

[B21] Healy M. W. Schexnayder B. Connell M. T. Terry N. DeCherney A. H. Csokmay J. M. (2016). Intrauterine adhesion prevention after hysteroscopy: a systematic review and meta-analysis. *Am. J. Obstet. Gynecol.* 215 267–275.e7. 10.1016/j.ajog.2016.05.001 27173082

[B22] Hellström M. Moreno-Moya J. M. Bandstein S. Bom E. Akouri R. R. Miyazaki K. (2016). Bioengineered uterine tissue supports pregnancy in a rat model. *Fertil. Steril.* 106 487–496.e1. 10.1016/j.fertnstert.2016.03.048 27068301

[B23] Hsu M. N. Liao H. T. Truong V. A. Huang K. L. Yu F. J. Chen H. H. (2019). CRISPR-based activation of endogenous neurotrophic genes in adipose stem cell sheets to stimulate peripheral nerve regeneration. *Theranostics* 9 6099–6111. 10.7150/thno.36790 31534539 PMC6735509

[B24] Huang Y. Wang J. Yang F. Shao Y. Zhang X. Dai K. (2017). Modification and evaluation of micro-nano structured porous bacterial cellulose scaffold for bone tissue engineering. *Mater. Sci. Eng. C Mater. Biol. Appl.* 75 1034–1041. 10.1016/j.msec.2017.02.174 28415386

[B25] Imafuku A. Oka M. Miyabe Y. Sekiya S. Nitta K. Shimizu T. (2019). Rat mesenchymal stromal cell sheets suppress renal fibrosis via microvascular protection. *Stem Cells Transl. Med.* 8 1330–1341. 10.1002/sctm.19-0113 31675167 PMC6877761

[B26] Jun I. Ahmad T. Bak S. Lee J. Y. Kim E. M. Lee J. (2017). Spatially assembled bilayer cell sheets of stem cells and endothelial cells using thermosensitive hydrogels for therapeutic angiogenesis. *Adv. Healthc. Mater.* 6:1601340. 10.1002/adhm.201601340 28230931

[B27] Kim S. Kim M. Jung S. Kwon K. Park J. Kim S. (2019). Co-delivery of therapeutic protein and catalase-mimic nanoparticle using a biocompatible nanocarrier for enhanced therapeutic effect. *J. Control Release* 309 181–189. 10.1016/j.jconrel.2019.07.038 31356840

[B28] Kim Y. Y. Park K. H. Kim Y. J. Kim M. S. Liu H. C. Rosenwaks Z. (2019). Synergistic regenerative effects of functionalized endometrial stromal cells with hyaluronic acid hydrogel in a murine model of uterine damage. *Acta Biomater.* 89 139–151. 10.1016/j.actbio.2019.03.032 30898731

[B29] Kuramoto G. Shimizu T. Takagi S. Ishitani K. Matsui H. Okano T. (2018). Endometrial regeneration using cell sheet transplantation techniques in rats facilitates successful fertilization and pregnancy. *Fertil. Steril.* 110 172.e–181.e. 10.1016/j.fertnstert.2018.03.007 29980256

[B30] Kuramoto G. Takagi S. Ishitani K. Shimizu T. Okano T. Matsui H. (2015). Preventive effect of oral mucosal epithelial cell sheets on intrauterine adhesions. *Hum. Reprod.* 30 406–416. 10.1093/humrep/deu326 25475585

[B31] Lee H. J. Choi B. Kim Y. Lee S. E. Jin H. J. Lee H. S. (2019). The upregulation of toll-like receptor 3 via autocrine IFN-β signaling drives the senescence of human umbilical cord blood-derived mesenchymal stem cells through JAK1. *Front. Immunol.* 10:1659. 10.3389/fimmu.2019.01659 31396213 PMC6665952

[B32] Li C. Ouyang L. Pence I. J. Moore A. C. Lin Y. Winter C. W. (2019). Buoyancy-driven gradients for biomaterial fabrication and tissue engineering. *Adv. Mater.* 31:e1900291. 10.1002/adma.201900291 30844123 PMC6606439

[B33] Li Z. Yan G. Diao Q. Yu F. Li X. Sheng X. (2019). Transplantation of human endometrial perivascular cells with elevated CYR61 expression induces angiogenesis and promotes repair of a full-thickness uterine injury in rat. *Stem Cell Res. Ther.* 10:179. 10.1186/s13287-019-1272-3 31215503 PMC6582612

[B34] Li X. Sun H. Lin N. Hou X. Wang J. Zhou B. (2011). Regeneration of uterine horns in rats by collagen scaffolds loaded with collagen-binding human basic fibroblast growth factor. *Biomaterials* 32 8172–8181. 10.1016/j.biomaterials.2011.07.050 21821282

[B35] Lin N. Li X. Song T. Wang J. Meng K. Yang J. (2012). The effect of collagen-binding vascular endothelial growth factor on the remodeling of scarred rat uterus following full-thickness injury. *Biomaterials* 33 1801–1807. 10.1016/j.biomaterials.2011.11.038 22136717

[B36] Liu F. Hu S. Wang S. Cheng K. (2019a). Cell and biomaterial-based approaches to uterus regeneration. *Regen. Biomater.* 6 141–148. 10.1093/rb/rbz021 31198582 PMC6547309

[B37] Liu F. Hu S. Yang H. Li Z. Huang K. Su T. (2019b). Hyaluronic acid hydrogel integrated with mesenchymal stem cell-secretome to treat endometrial injury in a rat model of Asherman’s syndrome. *Adv. Healthc. Mater.* 8:e1900411. 10.1002/adhm.201900411 31148407 PMC7045702

[B38] Logan P. C. Yango P. Tran N. D. (2018). Endometrial stromal and epithelial cells exhibit unique aberrant molecular defects in patients with endometriosis. *Reprod. Sci.* 25 140–159. 10.1177/1933719117704905 28490276 PMC6344981

[B39] Mao S. H. Chen C. H. Chen C. T. (2019). Osteogenic potential of induced pluripotent stem cells from human adipose-derived stem cells. *Stem Cell Res. Ther.* 10:303. 10.1186/s13287-019-1402-y 31623672 PMC6798413

[B40] Matsumura S. Ohta T. Yamanouchi K. Liu Z. Sudo T. Kojimahara T. (2017). Activation of estrogen receptor α by estradiol and cisplatin induces platinum-resistance in ovarian cancer cells. *Cancer Biol. Ther.* 18 730–739. 10.1080/15384047.2016.1235656 27689466 PMC5663418

[B41] Miki F. Maruyama T. Miyazaki K. Takao T. Yoshimasa Y. Katakura S. (2019). The orientation of a decellularized uterine scaffold determines the tissue topology and architecture of the regenerated uterus in rats. *Biol. Reprod.* 100 1215–1227. 10.1093/biolre/ioz004 30649202

[B42] Miyazaki K. Maruyama T. (2014). Partial regeneration and reconstruction of the rat uterus through recellularization of a decellularized uterine matrix. *Biomaterials* 35 8791–8800. 10.1016/j.biomaterials.2014.06.052 25043501

[B43] Mo X. Qin G. Zhou Z. Jiang X. (2019). Assessment of risk factors of intrauterine adhesions in patients with induced abortion and the curative effect of hysteroscopic surgery. *J. Invest. Surg.* 32 85–89. 10.1080/08941939.2017.1376130 28972429

[B44] Okano T. Yamada N. Sakai H. Sakurai Y. (1993). A novel recovery system for cultured cells using plasma-treated polystyrene dishes grafted with poly(N-isopropylacrylamide). *J. Biomed. Mater. Res.* 27 1243–1251. 10.1002/jbm.820271005 8245039

[B45] Owusu-Akyaw A. Krishnamoorthy K. Goldsmith L. T. Morelli S. S. (2019). The role of mesenchymal-epithelial transition in endometrial function. *Hum. Reprod. Update* 25 114–133. 10.1093/humupd/dmy035 30407544

[B46] Paim Á. Cardozo N. S. M. Pranke P. Tessaro I. C. (2018). Process system engineering methodologies applied to tissue development and regenerative medicine. *Adv. Exp. Med. Biol.* 1078 445–463. 10.1007/978-981-13-0950-2-23 30357637

[B47] Peng S. Zhang Y. Zhang J. Wang H. Ren B. (2010). ERK in learning and memory: a review of recent research. *Int. J. Mol. Sci.* 11 222–232. 10.3390/ijms11010222 20162012 PMC2821000

[B48] Qi Y. Lohman J. Bratlie K. M. Peroutka-Bigus N. Bellaire B. Wannemuehler M. (2019). Vitamin C and B3 as new biomaterials to alter intestinal stem cells. *J. Biomed. Mater. Res. A* 107 1886–1897. 10.1002/jbm.a.36715 31071241 PMC6626554

[B49] Rebelo A. R. Liu C. Schäfer K. H. Saumer M. Yang G. Liu Y. (2019). Poly(4-vinylaniline)/Polyaniline Bilayer-functionalized bacterial cellulose for flexible electrochemical biosensors. *Langmuir* 35 10354–10366. 10.1021/acs.langmuir.9b01425 31318565

[B50] Rogan H. Ilagan F. Tong X. Chu C. R. Yang F. (2020). Microribbon-hydrogel composite scaffold accelerates cartilage regeneration in vivo with enhanced mechanical properties using mixed stem cells and chondrocytes. *Biomaterials* 228:119579. 10.1016/j.biomaterials.2019.119579 31698227

[B51] Salazar C. A. Isaacson K. Morris S. (2017). A comprehensive review of Asherman’s syndrome: causes, symptoms and treatment options. *Curr. Opin. Obstet. Gynecol.* 29 249–256. 10.1097/GCO.0000000000000378 28582327

[B52] Santoso E. G. Yoshida K. Hirota Y. Aizawa M. Yoshino O. Kishida A. (2014). Application of detergents or high hydrostatic pressure as decellularization processes in uterine tissues and their subsequent effects on in vivo uterine regeneration in murine models. *PLoS One* 9:e103201. 10.1371/journal.pone.0103201 25057942 PMC4109986

[B53] Shadish J. A. Benuska G. M. DeForest C. A. (2019). Bioactive site-specifically modified proteins for 4D patterning of gel biomaterials. *Nat. Mater.* 18 1005–1014. 10.1038/s41563-019-0367-7 31110347 PMC6706293

[B54] Shum A. M. Che H. Wong A. O. Zhang C. Wu H. Chan C. W. (2017). A micropatterned human pluripotent stem cell-based ventricular cardiac anisotropic sheet for visualizing drug-induced arrhythmogenicity. *Adv. Mater.* 29:1602448. 10.1002/adma.201602448 27805726

[B55] Song T. Zhao X. Sun H. Li X. Lin N. Ding L. (2015). Regeneration of uterine horns in rats using collagen scaffolds loaded with human embryonic stem cell-derived endometrium-like cells. *Tissue Eng. Part A* 21 353–361. 10.1089/ten.TEA.2014.0052 25097004 PMC4292859

[B56] Sun H. Lu J. Li B. Chen S. Xiao X. Wang J. (2018). Partial regeneration of uterine horns in rats through adipose-derived stem cell sheets. *Biol. Reprod.* 99 1057–1069. 10.1093/biolre/ioy121 29931041

[B57] Vander Borght M. Wyns C. (2018). Fertility and infertility: definition and epidemiology. *Clin. Biochem.* 62 2–10. 10.1016/j.clinbiochem.2018.03.012 29555319

[B58] Wang B. Lv X. Chen S. Li Z. Yao J. Peng X. (2018). Use of heparinized bacterial cellulose based scaffold for improving angiogenesis in tissue regeneration. *Carbohydr. Polym.* 181 948–956. 10.1016/j.carbpol.2017.11.055 29254059

[B59] Wang X. Chen Z. Zhou B. Duan X. Weng W. Cheng K. (2018). Cell-sheet-derived ECM coatings and their effects on BMSCs responses. *ACS Appl. Mater. Interf.* 10 11508–11518. 10.1021/acsami.7b19718 29564888

[B60] Xia L. Meng Q. Xi J. Han Q. Cheng J. Shen J. (2019). The synergistic effect of electroacupuncture and bone mesenchymal stem cell transplantation on repairing thin endometrial injury in rats. *Stem Cell Res. Ther.* 10:244. 10.1186/s13287-019-1326-6 31391117 PMC6686409

[B61] Xiao B. Yang W. Lei D. Huang J. Yin Y. Zhu Y. (2019). PGS scaffolds promote the in vivo survival and directional differentiation of bone marrow mesenchymal stem cells restoring the morphology and function of wounded rat uterus. *Adv. Healthc. Mater.* 8:e1801455. 10.1002/adhm.201801455 30734535

[B62] Xin L. Lin X. Pan Y. Zheng X. Shi L. Zhang Y. (2019). A collagen scaffold loaded with human umbilical cord-derived mesenchymal stem cells facilitates endometrial regeneration and restores fertility. *Acta Biomater.* 92 160–171. 10.1016/j.actbio.2019.05.012 31075515

[B63] Xu H. L. Xu J. Zhang S. S. Zhu Q. Y. Jin B. H. ZhuGe D. L. (2017). Temperature-sensitive heparin-modified poloxamer hydrogel with affinity to KGF facilitate the morphologic and functional recovery of the injured rat uterus. *Drug Deliv.* 24 867–881. 10.1080/10717544.2017.1333173 28574291 PMC8241134

[B64] Xu L. Ding L. Wang L. Cao Y. Zhu H. Lu J. (2017). Umbilical cord-derived mesenchymal stem cells on scaffolds facilitate collagen degradation via upregulation of MMP-9 in rat uterine scars. *Stem Cell Res. Ther.* 8:84. 10.1186/s13287-017-0535-0 28420433 PMC5395893

[B65] Yang H. Wu S. Feng R. Huang J. Liu L. Liu F. (2017). Vitamin C plus hydrogel facilitates bone marrow stromal cell-mediated endometrium regeneration in rats. *Stem Cell Res. Ther.* 8:267. 10.1186/s13287-017-0718-8 29157289 PMC5697119

[B66] Yu J. Wang M. Y. Tai H. C. Cheng N. C. (2018). Cell sheet composed of adipose-derived stem cells demonstrates enhanced skin wound healing with reduced scar formation. *Acta Biomater.* 77 191–200. 10.1016/j.actbio.2018.07.022 30017923

[B67] Zhang S. S. Xia W. T. Xu J. Xu H. L. Lu C. T. Zhao Y. Z. (2017). Three-dimensional structure micelles of heparin-poloxamer improve the therapeutic effect of 17β-estradiol on endometrial regeneration for intrauterine adhesions in a rat model. *Int. J. Nanomed.* 12 5643–5657. 10.2147/IJN.S137237 28848344 PMC5557621

[B68] Zhang S. S. Xu X. X. Xiang W. W. Zhang H. H. Lin H. L. Shen L. E. (2020). Using 17β-estradiol heparin-poloxamer thermosensitive hydrogel to enhance the endometrial regeneration and functional recovery of intrauterine adhesions in a rat model. *FASEB J.* 34 446–457. 10.1096/fj.201901603RR 31914682

[B69] Zhang Y. Lin X. Dai Y. Hu X. Zhu H. Jiang Y. (2016). Endometrial stem cells repair injured endometrium and induce angiogenesis via AKT and ERK pathways. *Reproduction* 152 389–402. 10.1530/REP-16-0286 27486270

[B70] Zhang Z. Zhang D. Dou M. Li Z. Zhang J. Zhao X. (2016). Dendrobium officinale kimura et migo attenuates diabetic cardiomyopathy through inhibiting oxidative stress, inflammation and fibrosis in streptozotocin-induced mice. *Biomed. Pharmacother.* 84 1350–1358. 10.1016/j.biopha.2016.10.074 27802903

[B71] Zhao G. Cao Y. Zhu X. Tang X. Ding L. Sun H. (2017). Transplantation of collagen scaffold with autologous bone marrow mononuclear cells promotes functional endometrium reconstruction via downregulating ΔNp63 expression in Asherman’s syndrome. *Sci. China Life Sci.* 60 404–416. 10.1007/s11427-016-0328-y 27921235

[B72] Zhao G. Huang B. L. Rigueur D. Wang W. Bhoot C. Charles K. R. (2018). CYR61/CCN1 regulates sclerostin levels and bone maintenance. *J. Bone Miner. Res.* 33 1076–1089. 10.1002/jbmr.3394 29351359 PMC6002906

